# Building the Future of Clinical Diagnostics: An Analysis of Potential Benefits and Current Barriers in CRISPR/Cas Diagnostics

**DOI:** 10.1021/acssynbio.4c00816

**Published:** 2025-01-29

**Authors:** Jeanne E. van Dongen, Loes I. Segerink

**Affiliations:** BIOS Lab on a Chip Group, MESA+ Institute for Nanotechnology, Technical Medical Centre, Max Planck Institute for Complex Fluid Dynamics, University of Twente, P.O. Box 217, 7500 AE Enschede, The Netherlands

## Abstract

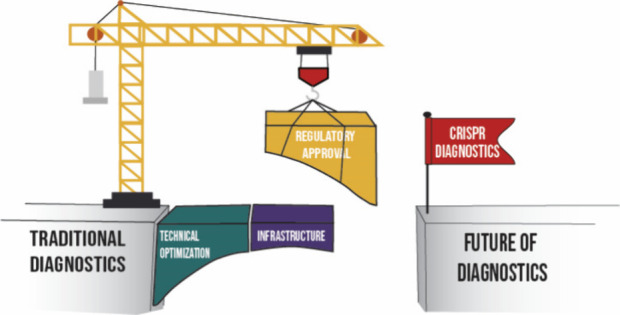

Advancements in molecular diagnostics, such as polymerase chain reaction and next-generation sequencing, have revolutionized disease management and prognosis. Despite these advancements in molecular diagnostics, the field faces challenges due to high operational costs and the need for sophisticated equipment and highly trained personnel besides having several technical limitations. The emergent field of CRISPR/Cas sensing technology is showing promise as a new paradigm in clinical diagnostics, although widespread clinical adoption remains limited. This perspective paper discusses specific cases where CRISPR/Cas technology can surmount the challenges of existing diagnostic methods by stressing the significant role that CRISPR/Cas technology can play in revolutionizing clinical diagnostics. It underscores the urgency and importance of addressing the technological and regulatory hurdles that must be overcome to harness this technology effectively in clinical laboratories.

## Introduction

Over the last century, clinical diagnostics has dramatically transformed patient care by introducing diagnostic tools like polymerase chain reaction (PCR) and next-generation sequencing (NGS). Quantitative PCR (qPCR), for example, has become the gold standard in nucleic acid-based diagnostics and found applications across various medical fields, including infectious diseases, cancer, and genetic disorders.^1^

However, advancements in personalized medicine,^2^ along with the emerging fields of liquid biopsy^3^ and point-of-care (PoC) testing,^4^ are expected to be more prominent in the clinic in the next century.^5^ Established molecular diagnostic techniques will face challenges here. Techniques like qPCR and NGS are costly, require sophisticated equipment, and demand highly trained personnel.^6^ These factors can limit their accessibility and scalability, particularly in resource-limited settings. Besides that, also technical limitations exist, such as the length of target sequences that can be effectively analyzed and the ability to identify subtle genetic variations, like single nucleotide polymorphism (SNP) changes or epigenetic modifications.

Therefore, there is a growing need for innovative diagnostic approaches to address these upcoming demands. Initially celebrated for its precision in gene editing, CRISPR/Cas technology was adapted to enable precise nucleic acid detection.^7−10^ While current research and development increasingly focus on PoC applications,^11^ the utility of CRISPR/Cas extends well beyond its ability to integrate into on-site diagnostic tools. However, despite this promise, clinical laboratories’ actual adoption of CRISPR-based diagnostics is limited, if not absent.

In this perspective paper, we explore the potential of integrating CRISPR/Cas technology into clinical diagnostics. Despite its current adoption challenges, we propose that CRISPR/Cas diagnostics (CRISPRDx) could revolutionize the clinical diagnostic field. First, we introduce CRISPR/Cas proteins as molecular biosensors, followed by examining specific cases where CRISPR/Cas technology addresses diagnostic challenges. We end with discussing the technological, regulatory, and ethical hurdles one must overcome to harness CRISPRDx effectively.

## CRISPR/Cas Proteins as Molecular Biosensors

**Figure 1:**
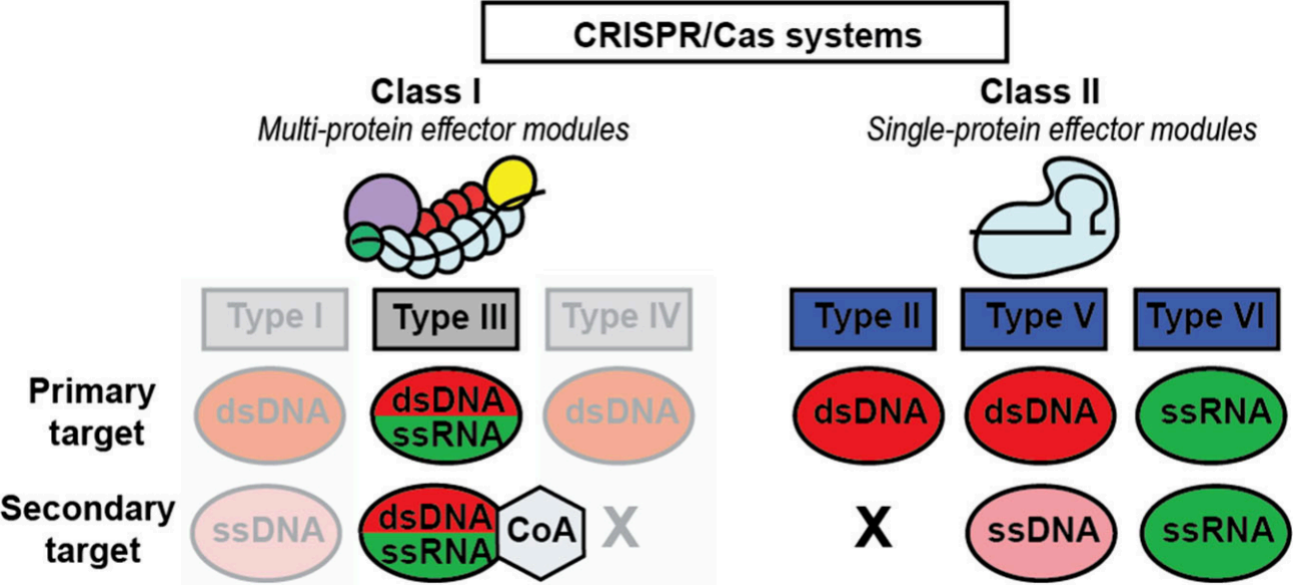
Classification of CRISPR/Cas systems is based on two classes defined by their crRNA–effector protein complex. The six types are distinguished by the presence of a signature protein and their corresponding subtypes.

CRISPRDx relies on programmable Cas enzymes, guided by CRIPSR RNAs (crRNAs), to target and cleave specific nucleic acid sequences. These systems are categorized into classes^12^ based on their effector proteins and cleavage mechanisms (Figure 1).

### Class I CRISPR/Cas Systems

Class I CRISPR/Cas systems are less frequently used in diagnostics because of their complexity. These systems use a multiprotein complex called cascade to identify and bind to target nucleotide sequences. The only class I CRISPR/Cas systems currently used for biosensing are the type III-A systems like MORIARTY^13^ and SCOPE.^14^ These systems comprise complexes of multiple proteins that function together to both recognize and cleave RNA molecules. The recognition of the target RNA activates the large subunit of the cascade—the so-called CRISPR polymerase—which cleaves DNA and produces small cyclic oligonucleotides (CoAs). These CoAs act as signaling molecules to activate auxiliary effectors. This can, for example, be nonspecific RNases that can cleave single-stranded RNA (ssRNA) fluorophore quencher pairs to enable fluorescent read-out. Additionally, the type III-A systems produce protons (H^+^) and pyrophosphate upon target recognition, which can also be used in colorimetric and fluorometric detection methods, offering multiple read-out options for diagnostic applications.^15^

### Class II CRISPR/Cas Systems

Class II CRISPR/Cas systems are preferred for *in vitro* diagnostics due to their simplified, single-protein effector modules. These systems can roughly be divided into two categories: the types that perform targeted cleavage only (like Cas9, type II) and those that, exhibit an additional nontargeted collateral cleavage activity (types V and VI).

The first category is mostly used as the enzymatic deactivated version (dCas9), which retains the ability to bind double-stranded DNA (dsDNA) without cleaving it, making them versatile tools for diagnostics through sequence recognition.^16−18^ However, the other category, comprising type V (Cas12) and type VI (Cas13) systems that target DNA and RNA, respectively, exhibit collateral cleavage activity, greatly improving their detection limit as this cleavage is multiturnover. This means that a single effector protein can cleavage thousands of nucleotides upon recognizing a single target molecule. This collateral cleavage is harnessed in diagnostic methods like SHERLOCK,^19^ DETECTR,^20^ and HOLMES,^21^ where either single-stranded DNA (ssDNA) or ssRNA labeled with a fluorophore and quencher serve as reporter molecules. When the target sequence is present, cleavage of these labeled nucleotides releases a fluorescent signal.

## Specific Cases Where CRISPR/Cas Technology Overcomes Limitations

The freedom to choose a combination of Cas selection and custom designing of crRNA sequences enables researchers to target various nucleotide sequences. However, using CRISPR/Cas technology will not always be beneficial over current diagnostic methods. Here, we discuss several user cases where using CRISPR-based sensing will be beneficial, improving diagnostic ease and/or accuracy.

### Rapid PoC Diagnostics during Viral Outbreaks

During the COVID-19 pandemic, the SHERLOCK^22,23^ and DETECTR^24,25^ platforms, among others, demonstrated their capability to detect SARS-CoV-2 with high sensitivity and specificity. Developed by Sherlock Biosciences, SHERLOCK uses the collateral cleavage activity of Cas13 to produce a fluorescent signal upon detecting the virus’s RNA. Similarly, DETECTR, developed by Mammoth Biosciences, employs Cas12 for rapid detection. Just like other (non-commercial) CRISPR-based SARS-CoV-2 tests, SHERLOCK and DETECTR offer high-throughput testing capabilities that match the accuracy of PCR tests but at faster speeds (typically within 1–2 h compared to several hours for the full PCR work-flow), with 95% positive predictive agreement and 100% negative predictive agreement.^24,26−28^ The real PoC performance of CRISPRDx is also demonstrated with these assays, showing these results in assay times of less than 1 h while operated at room or body temperature and without the need for sophisticated laboratory equipment.^29,30^

However, important to note that while SHERLOCK and DETECTR represent significant proof-of-concept advances, they have not been fully realized as practical PoC solutions in real-world healthcare environments. Operational complexities, such as preamplification steps and manual handling, restrict their deployment outside of well-equipped laboratories. For instance, the SHERLOCK assay, while marketed as a PoC solution, is only FDA-approved for use in Clinical Laboratory Improvement Amendments (CLIA)-certified facilities due to its operational complexity.^31^ This underscores the gap between laboratory demonstrations and practical deployment.^31^

Besides PoC benefits, CRISPR-based diagnostics can rapidly and accurately identify pathogens such as viruses and bacteria by detecting their unique genetic material.^32,33^ Additionally, the straightforward programmability of CRISPR via crRNA design allows for rapid adaptation to emerging diseases, as seen during the early stages of the COVID-19 pandemic, where timely diagnosis is critical.^34^ Due to the high selectivity toward a target, CRISPR/Cas systems also excel at differentiating closely related SARS-CoV-2 strains with 100% specificity,^35^ essential for effective infectious disease management.

### Low-Abundant Mutation Detection

One of the advantages advertised for CRISPRDx is their ability to detect or differentiate SNPs. However, this is more complex than it sounds: CRISPR systems exhibit varying sensitivity to mismatches between their crRNA and the target DNA, related to Cas proteins’ structural and functional differences. For example, CRISPR/Cas9 is generally tolerant toward mismatches, whereas variants like SpCas9-HF1 were engineered to enhance mismatch sensitivity and reduce off-target effects over the entire recognition site.^36^ By contrast, type III-A CRISPR systems like MORIARTY and SCOPE have mismatch sensitivity that depends on the position of the mismatch relative to a certain subunit.^13,14^

Also, the position of the mismatch compared to the crRNA architecture is of great importance. Mismatches in the “seed region” of the crRNA, a specific short sequence critical for initiating the binding of the CRISPR complex to the target nucleotide, can significantly impact the binding and cleavage efficiency of Cas proteins.^7,37,38^ However, the precise catalytic effect of a mismatch is different for every crRNA-Cas pair, and therefore requires significant assay calibration, hindering the rapid development of CRISPRDx assays.

However, once rightly optimized, CRISPR’s single nucleotide specificity helps detect rare genetic mutations that traditional PCR might miss. For example, Cas12a, in combination with PCR, can detect SNPs down to 0.5%, particularly for mutations close to the protospacer adjacent motif (PAM) site.^39^ Predictive tools like ARTEMIS facilitate this process by identifying targetable SNPs in PAM-proximal seed regions genome-wide, making them ideal candidates for CRISPR-based detection.^40^ By reducing the need for labor-intensive testing, these tools accelerate assay development.^39^ However, suppose one wants to target specific SNPs located further from the PAM site. In that case, optimizing the number of PCR cycles has been shown to lower the detection limit to around 3–6% of a simulated tumor fraction.^38,41^ Besides Cas12a, also other CRISPR systems like Cas14a, combined with blocker displacement amplification, offer susceptible and accurate detection of SNPs, identifying cancer-related mutations with as low as 0.1% variant allele frequency, demonstrating superior performance in low abundant mutation detection.^42^

Beyond pathogen and mutation detection, CRISPRDx is further expanding its diagnostic capabilities by monitoring gene expression patterns and identify epigenetic modifications on the single nucleotide level.^43−45^ This makes CRISPR valuable in areas like cancer diagnostics, where identifying rare mutations is crucial for targeted therapies.^46^

### Detection of Short or Fragmented Nucleotides

**Figure 2:**
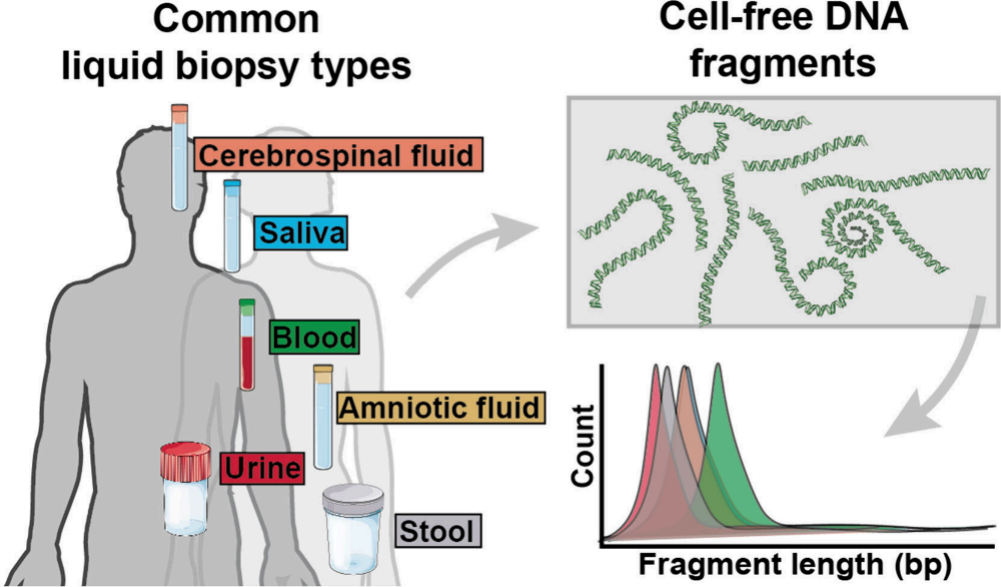
DNA fragmentation in liquid biopsies is influenced by nucleosome organization, chromatin structure, gene expression, and nuclease content of the tissue of origin.

Liquid biopsies are increasingly utilized in clinical settings because they are less invasive and could provide real-time molecular insights. This makes liquid biopsies a valuable alternative to tissue biopsies in areas like prenatal testing, oncology, and organ transplant monitoring.^3,48^ However, a real challenge in liquid biopsy analysis is the size distribution of DNA fragments, which is influenced by nucleosome organization, chromatin structure, and tissue-specific nuclease activity. Also, the origin of the biopsy plays a role. Sources, like saliva, stool, urine, cerebrospinal fluid, and amniotic fluid, exhibit varying fragment sizes influenced by their biological environments (Figure 2). For example, blood, including plasma and serum, typically shows a bimodal distribution of DNA fragments with peaks around 166 base pairs (bp) and smaller fragments below 100 bp.^49^ Detecting short or fragmented nucleotides poses a significant challenge for PCR-based methods, particularly for ultrashort circulating tumor DNA (usctDNA)^50^ and microRNAs,^51^ which with typical lengths of 20–60 bp often fall below the amplification limits of standard PCR techniques that require amplicon sizes varying between 75 and 150 bp for quantitative analysis. CRISPR/Cas systems use guide RNAs with spacer regions typically 20–45 nucleotides long, therefore enabling precise recognition of highly fragmented nucleic acids.^52,53^ However, as we will discuss in the Limit of Detection of CRISPRDx and Enzyme Kinetics section, current enzyme kinetics and signal detection constraints prevent CRISPR-based diagnostics from achieving amplification-free detection at subfemtomolar (sub-fM) concentrations—a critical requirement given the typically low concentrations of nucleic acids in liquid biopsy samples.

### Unprocessed Sample Analysis

CRISPR/Cas-based assays enable the direct analysis of complex biological samples without extensive nucleic acid extraction and purification. While most assays still require some form of sample preparation, such as lysis or pretreatment, before CRISPR analysis can be performed, literature has demonstrated that CRISPR can be directly applied to a variety of sample types, such as blood,^54−56^ serum,^57^ saliva,^58,59^ urine,^60^ and sweat.^61^ This significantly simplifies the workflow and reduces testing time, making these assays ideal for rapid diagnostics in clinical settings.

However, direct application of CRISPRDx to raw samples can introduce trade-offs in sensitivity and performance. To bypass the effects of inhibitors or nonspecific interactions in unprocessed samples, they are often diluted within CRISPR reaction buffers to minimize these effects.^61^ This dilution, however, can compromise sensitivity compared to workflows using extracted or purified nucleic acids.^62^ Despite these challenges, direct application simplifies workflows and reduces testing time, making CRISPR-based assays promising tools for rapid diagnostics in clinical settings. The continued development of methods to mitigate sensitivity loss while maintaining the benefits of unprocessed sample analysis will be critical for expanding the clinical usability of CRISPR diagnostics.

## Current Hurdles in Adoption

CRISPRDx provide several advantages over traditional PCR and sequencing methods, particularly regarding operational temperature, adaptability cost, simplicity, and specificity. These attributes make CRISPR/Cas technology a strong candidate for various diagnostic applications where rapid and accurate results are crucial. While CRISPR/Cas technology has demonstrated significant potential across various diagnostic applications, the path to widespread clinical adoption is challenging, as highlighted in the user cases. Key issues such as the test duration, detection limits for many genetic tests, standardization, and ensuring compatibility with existing clinical workflows must be addressed before CRISPRDx can become a routine part of clinical practice.

### Limit of Detection of CRISPRDx and Enzyme Kinetics

**Figure 3:**
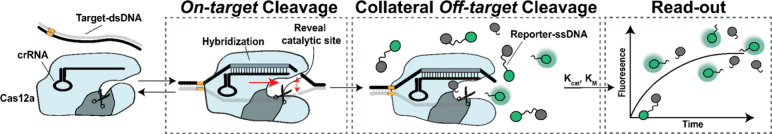
DNA detection scheme using CRISPR/Cas12a. The Cas12a-crRNA complex specifically binds to a target DNA sequence adjacent to a PAM, activating its catalytic site for on-target cleavage. This activation also induces indiscriminate *trans*-cleavage of nearby ssDNA reporter molecules labeled with a quencher and fluorophore.

CRISPRDx detects target DNA/RNA through protein–RNA complexes that bind double-stranded sequences, triggering specific on-target cleavage and slower, multiturnover *trans*-cleavage of single-stranded nucleotide reporters (Figure 3). However, the low catalytic rate of *trans*-cleavage (*k*_cat_/*K*_M_ = 10^5^–10^6^ M^–1^ s^–1^)^63,64^ and (fluorescent) signal dilution in larger sample volumes limit detection sensitivity to picomolar (pM) levels without preamplification.^65^

Reported attomolar (aM) detection levels in CRISPRDx typically depend on preamplification methods like PCR, loop-mediated isothermal amplification (LAMP), or recombinase polymerase amplification (RPA), which increase sensitivity but add complexity and risk compromising quantitative accuracy. Given that many amplification reactions are already specific, the key question becomes when does the CRISPR step provide added value? In cases such as detecting highly abundant targets or identifying pathogens with distinct genomes (e.g., influenza vs SARS-CoV-2), where small sequence variations do not impact detection—traditional amplification techniques or simpler detection methods may suffice without the added complexity of CRISPR-based systems. On the other hand detecting challenging targets such as SNPs or closely related sequences, particularly in PoC settings requiring speed and precision will benefit from CRISPR’s programmability and selectivity.

Integrating CRISPR/Cas systems with microfluidics presents a promising solution to overcome the limitations of preamplification. Sample partitioning into small chambers or droplets can theoretically improve detection limits from pM to aM concentrations, depending on partition size and number.^66−69^ However, traditional digital CRISPR sensors often fail to detect single molecules in microsized droplets due to a combination of enzyme kinetics and sensitivity issues such as reporter dilution.^70−75^ These challenges can sometimes be mitigated by in-droplet isothermal amplification, though this requires complex synchronization of amplification and CRISPR activity.

Achieving amplification-free, subfemtomolar detection—essential for applications like liquid biopsy diagnostics—requires innovations such as enhanced *trans*-cleavage efficiency in CRISPR proteins or cascades to amplify signals without nucleic acid amplification.^76−78^ These advancements could bridge the gap between laboratory techniques and clinical diagnostics, enabling CRISPRDx to reach its full potential.

### Technical and Logistical Challenges

**Figure 4:**
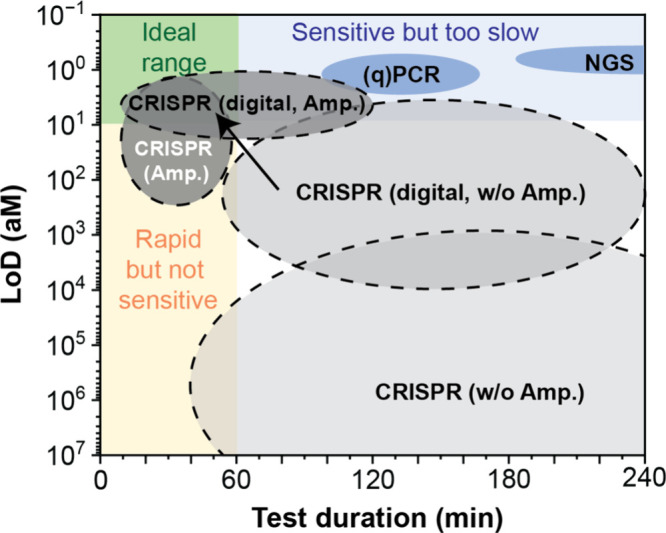
Comparison of the LoD and test durations of the gold standard method (qPCR), NGS, and CRISPR/Cas assays. The four major classes of CRISPR-based methods (without amplification, with amplification, droplet-based without amplification, and droplet-based with amplification) are shown as separate shaded areas, providing a clear distinction between their respective performance ranges. Adapted from ref (106). Copyright 2022 Elsevier Ltd.

To achieve widespread adoption, CRISPRDx must demonstrate clear advantages in speed and detection limit (Figure 4 and Table 1). To evaluate the performance of CRISPRDx, we adopted the figure of merit (FoM), defined as the product of the limit of detection (LoD, aM) and the CRISPR reaction time (*T*, minutes).^79^ This metric provides a quantitative measure of the trade-off between sensitivity and speed, with a smaller FoM indicating a method capable of detecting smaller target quantities more quickly. By minimizing the FoM, we can identify and prioritize strategies that enhance diagnostic performance.

**Table 1:** Comparison of CRISPRDx with qPCR and NGS Using Data from Reference (79) and Referenced Studies^a^

method	amplification	amp. time (min)	reaction time (min)	LoD (aM)	FoM(aM·min)	ref
CRISPR (w/o Amp.)	none	none	15–1440	1 × 10^3^–3.7 × 10^9^	3.0 × 10^4^–7.4 × 10^9^	(87)–^91^
CRISPR (Amp.)	LAMP, RPA, PCR	10–50	5–60	1.6–83	30–7.2 × 10^3^	(8), (92)–^95^
CRISPR (digital w/o Amp.)	none	none	5–60	8.3–1 × 10^4^	124–5 × 10^4^	(92), (93), (96), (97)
CRISPR (digital Amp.)	RPA	*(one pot)	50–120	1.5–23	90–2.7 × 10^3^	(98)–^101^
qPCR (typical)	yes	∼90–180	N/A	*1–5 copies	3 × 10^4^–1.2 × 10^5^	(102), (103)
NGS	yes	∼180–600	N/A	1% allele frequency	2.4 × 10^4^–6 × 10^5^	(104), (105)

a Key metrics include amplification methods, amplification time, reaction time, LoD, and FoM. These data were used to construct Figure 4.

While CRISPRDx, particularly when combined with preamplification or digitalization, outperforms PCR in certain FoM measures (Table 1 and Figure 4), qPCR remains the clinical gold standard due to its reliability, established infrastructure, and superior capacity for multiplexing through probe design.^80^ For example, during the COVID-19 pandemic, PCR emerged as the dominant technology,^81^ despite the emergence of numerous CRISPR-based tests, including SHERLOCK and DETECTR.^30,70,82−85^ This can largely be attributed to CRISPRDx’s lower throughput: While qPCR is well-suited for high-throughput workflows, capable of processing large sample volumes simultaneously, CRISPRDx often requires preamplification and manual (pipetting) steps, complicating workflows and reducing scalability. Additionally, multiplexing in CRISPRDx assays remains in its early stages, further restricting their application in scenarios requiring simultaneous detection of multiple targets.^86^

Cost-effectiveness and ease of use are also critical factors influencing CRISPRDx adoption. Technical challenges remain a significant barrier to using CRISPR/Cas-based diagnostics, particularly in PoC and home-use settings. Most CRISPRDX assays require sample processing steps like lysis or pretreatment. Furthermore, there is a need for ultralow temperature storage to keep the CRISPR proteins intact which complicate use in resource-limited environments. Together, these requirements often limit accessibility and scalability, especially in areas without well-equipped laboratories or trained personnel.^81^ Addressing these barriers is critical to transitioning CRISPRDx from proof-of-concept demonstrations to fully deployable PoC solutions.

### Stakeholder Perspectives

Ideally, by reducing the time from diagnosis to treatment CRISPRDx should streamline treatment, especially in critical areas like oncology and infectious diseases. However, its adoption may face challenges similar to those encountered by digital droplet PCR (ddPCR). Despite offering superior sensitivity and quantification for nucleic acid detection, ddPCR remains underutilized due to high costs, the need for specialized training, complex workflows, and a lack of standardized protocols and cross-laboratory validation.^107−109^ Therefore, for CRISPRDx to gain broader acceptance, it must address areas like costs, standardized workflows validated across multiple centers and focus on streamlined integration into existing clinical infrastructure. Eventually, to achieve widespread clinical adoption, clinicians need confidence in the reliability and consistency of CRISPR tests to ensure accurate results across conditions.

Patients are likely to benefit from CRISPR’s enhanced diagnostic capabilities, particularly in speed of diagnostic results. However, these advancements raise ethical concerns about privacy and data security. Similar to controversies in direct-to-consumer genetic testing—such as nonconsensual ancestry tracing^110^ and insurance discrimination^111^—CRISPRDx generate sensitive genetic information that could be misused. To build trust, developers of CRISPRDx must prioritize robust data protection measures and comply with regulations like the General Data Protection Regulation in the European Union^112^ and the Health Insurance Portability and Accountability Act in the United States,^113^ ensuring ethical and secure handling of genetic data. Although we believe the regulatory process for CRISPRDx may be more straightforward than therapeutic CRISPR applications, regulatory bodies might still create a complex approval landscape for CRISPR-based diagnostics. In the United States, CRISPRDx most likely must follow the FDA’s regulatory pathways for *in vitro* diagnostics (IVDs). However, more rigorous premarket approval (PMA) might be required for high-risk disease detection. For instance, Mammoth Biosciences’ DETECTR Reagent Kit and DETECTR BOOST SARS-CoV-2 Reagent Kit both received EUA, showcasing CRISPRDx as a scalable and efficient solution during public health emergencies. However, these platforms have not yet achieved full regulatory approval for permanent clinical use. In the European Union, CRISPRDx are subject to the *In Vitro* Diagnostic Regulation (IVDR), which imposes stringent oversight, particularly for high-risk applications. The IVDR necessitates extensive clinical evidence and continuous postmarket surveillance, posing additional hurdles for widespread adoption, and might, therefore, be more stringent than approval in the United States.

For industry players commercializing CRISPRDx, the focus is most probably on scalability and profitability. Demonstrating clear advantages over qPCR methods in speed, accuracy, and cost-effectiveness is essential for market adoption. Additionally, navigating the complex intellectual property landscape is crucial. Key patents held by Sherlock Biosciences and Mammoth Biosciences, particularly on Cas12-based technologies, may restrict specific commercial applications, particularly in the United States.

## Future Perspectives and Research Directions

We believe the future of CRISPR/Cas-based diagnostics lies in not only replacing existing methods but also enabling new possibilities, such as detecting rare mutations with 0.1% allele frequency, analyzing unprocessed samples, and identifying fragmented nucleic acids crucial for liquid biopsies. CRISPR also offers real-time epigenetic monitoring^114−116^ and rapid adaptation to new pathogens, unmatched by traditional methods.

However, advancing CRISPRDx requires enhancing detection limits without preamplification, integrating seamlessly into clinical workflows, and developing fast, single-step assays adaptable to existing infrastructure. For PoC applications, focus must align with WHO’s ASSURED criteria, prioritizing affordability, robustness, and minimal infrastructural needs.

Overcoming these challenges will allow CRISPR/Cas systems to transform clinical diagnostics, improving patient outcomes and global healthcare delivery. Ultimately, CRISPR technology is poised to become a cornerstone of future diagnostic methodologies, driving innovation and enhancing the quality of diagnosis inside and outside the hospital.
